# Epidemiology of Doublet/Multiplet Mutations in Lung Cancers: Evidence that a Subset Arises by Chronocoordinate Events

**DOI:** 10.1371/journal.pone.0003714

**Published:** 2008-11-13

**Authors:** Zhenbin Chen, Jinong Feng, Carolyn H. Buzin, Steve S. Sommer

**Affiliations:** Department of Molecular Genetics, City of Hope National Medical Center, Duarte, California, United States of America; Ohio State University Medical Center, United States of America

## Abstract

**Background:**

Evidence strongly suggests that spontaneous doublet mutations in normal mouse tissues generally arise from chronocoordinate events. These chronocoordinate mutations sometimes reflect “mutation showers”, which are multiple chronocoordinate mutations spanning many kilobases. However, little is known about mutagenesis of doublet and multiplet mutations (domuplets) in human cancer. Lung cancer accounts for about 25% of all cancer deaths. Herein, we analyze the epidemiology of domuplets in the *EGFR* and *TP53* genes in lung cancer. The *EGFR* gene is an oncogene in which doublets are generally driver plus driver mutations, while the *TP53* gene is a tumor suppressor gene with a more typical situation in which doublets derive from a driver and passenger mutation.

**Methodology/Principal Findings:**

*EGFR* mutations identified by sequencing were collected from 66 published papers and our updated *EGFR* mutation database (www.egfr.org). *TP53* mutations were collected from IARC version 12 (www-p53.iarc.fr). For *EGFR* and *TP53* doublets, no clearly significant differences in race, ethnicity, gender and smoking status were observed. Doublets in the *EGFR* and *TP53* genes in human lung cancer are elevated about eight- and three-fold, respectively, relative to spontaneous doublets in mouse (6% and 2.3% versus 0.7%).

**Conclusions/Significance:**

Although no one characteristic is definitive, the aggregate properties of doublet and multiplet mutations in lung cancer are consistent with a subset derived from chronocoordinate events in the *EGFR* gene: i) the eight frameshift doublets (present in 0.5% of all patients with *EGFR* mutations) are clustered and produce a net in-frame change; ii) about 32% of doublets are very closely spaced (≤30 nt); and iii) multiplets contain two or more closely spaced mutations. *TP53* mutations in lung cancer are very closely spaced (≤30 nt) in 33% of doublets, and multiplets generally contain two or more very closely spaced mutations. Work in model systems is necessary to confirm the significance of chronocoordinate events in lung and other cancers.

## Introduction

Cancer is a heterogeneous, multi-step, complex genetic disease resulting from accumulated mutations. Cancers generally were considered to arise by the accumulation of an estimated 5–7 causative mutations [1], [2]. Recently, however, Vogelstein and others have shown in breast and colorectal cancers that individual tumors accumulate ∼90 somatically mutated genes [3]. However, a much smaller number (estimated to be ∼11) are likely to be involved in tumorigenesis in any given tumor. The accumulation of so many contributory mutations is hard to explain solely by the frequency of multiple independent mutation events. The accumulation of mutations in certain cancers may reflect a mutator phenotype with globally random mutations [4]–[6]. Sequential selection offers another explanation [7], [8]. One mutation causes over-replication, giving rise to a clone, which further over-replicates due to a second mutation, etc. Recently the existence of mutation showers were reported, raising the possibility of “cancer in an instant” if scattered mutation showers occur. Mutation showers were found after analyses of intragenic doublet mutations revealed that they were clustered and chronocoordinate [9].

We demonstrated that the majority of doublets in the *EGFR* gene have a different mutation pattern relative to singlets and consist of driver plus driver mutations, due to sequential or chronocoordinate mutations, putatively followed by functional selection of two individually sub-optimal mutations [10]. In addition, acquired second mutations (e.g. T790M) in four *EGFR* doublets have been reported after treatment with gefitinib and erlotinib and associated with drug resistance and disease relapse [11], [12]. In contrast, most *TP53* and *lacI* doublets show driver plus passenger mutations in lung cancers and in normal Big Blue mouse tissue, respectively. However, the epidemiology and mechanisms underlying mutagenesis of doublets and multiple mutations are unclear.

To elucidate the frequency of doublets and the distinct mutation pattern and spectrum in the *EGFR* gene, the epidemiology of doublet mutations in lung cancer is analyzed and compared to that of doublets in the *TP53* gene. Herein, we find that smoking status, race, ethnicity, gender, and age are not risk factors for doublet occurrence in the *EGFR* and *TP53* genes. The relative frequency of doublets is higher in human lung cancer than in normal mouse tissue. The existence of OMIDI pairs (see Terminology and Abbreviations in Materials and Methods) in *EGFR*, the high frequency of allelic versus compound heterozygote doublets, the high frequency of closely spaced doublets, and the clustering in multiplets are consistent with chronocoordinate mutations contributing to at least a subset of the *EGFR* and *TP53* doublet and multiplet mutations found in human lung cancer.

## Results

### Epidemiology of doublet mutations in the EGFR and TP53 genes in lung cancer

Analysis by smoking status, gender, and ethnicity does not reveal a significant difference in doublet frequency in the *EGFR* gene (Table 1, Table S1). The average age of patients with singlets and doublets is similar (61.4±11.3 *vs* 65.8±10.3).

**Table 1:** The epidemiology of *EGFR* and *TP53* doublets versus singlets^1^ in lung cancer.

	Singlets	Doublets 4
*EGFR* 2		
Non-smoker	296	22 (6.9%)
Smoker	314	12 (3.7%)
NA	916	62
Female	431	58 (11.9%)
Male	289	28 (8.8%)
NA	806	10
Asian	727	77 (9.6%)
Caucasian	104	11 (9.6%)
NA	695	8
Average age	61.4±11.3	65.8±10.3
*TP53* 3		
Non-smoker	192	2 (1.0%)
Smoker	877	11 (1.2%)
NA	1257	41
Female	382	4 (1.0%)
Male	841	22 (2.5%)
NA	1103	28
Asian	720	24 (3.2%)
Caucasian	1443	26 (1.8%)
NA	163	4
Average age	63.1±10.6	62.8±9.0

1 Total number of mutations: *EGFR*, n = 1627; *TP53*, n = 2387.

2 The number of singlets, doublets and multiplets in *EGFR* is 1526, 98 and 5, respectively

3 The number of singlets, doublets and multiplets in *TP53* is 2326, 54 and 7, respectively

4 The number and percentage of doublets among the total singlets+doublets in each category by row.

NA: not available.

The results for the *TP53* gene in lung cancer are similar with one exception. The frequency of *TP53* doublets is significantly higher in Asians than in Caucasians (3.2% *vs* 1.8%, P = 0.03) (Table 1, Table S2), but this is not significant when corrected for multiple comparison (six Fisher's Exact Tests performed for the *EGFR* and *TP53* genes). Additional data are necessary to evaluate this point.

Overall, 98/1627 (6.0%) of EGFR mutations and 54/2387 (2.3%) of TP53 mutations in human lung cancers are doublets, a much higher frequency than that observed in the *lacI* gene in Big Blue mice (8-fold and 3-fold, respectively) (Table 2).

**Table 2:** A comparison of doublet characteristics among *EGFR* (lung cancer), *LacI* (Big Blue) and *TP53* (lung cancer).

Parameter	*EGFR*	*LacI*	*TP53*
**Frequency**			
# of doublets	98	51	54
Percentage of mutations that are doublets	6% (98/1627)	0.7% (51/7247)	2.3% (54/2387)
**Spectrum**			
Spectrum of singlets vs. doublets	Different (P<0.00001)	Similar	Similar
# of total nonsense mutations	0.2% (3/1627)	14.2% (1028/7247)	8.9% (212/2387)
# of singlet MIDIs	830	1038	271
% of singlet MIDIs that are IMIDIs	99.5% (826/830)	13.8% (143/1038)	14.8% (40/271)
% of doublets with precise recurrences	46.9% (45/96)	0.0%	0.0%
% of doublets with precise recurrences (3 or more identical events)	38.5% (37/96)	0.0%	0.0%
% of doublets containing at least one mutation seen in at least three doublets	75% (72/96)	2% (1/51)	1.9% (1/54)
# of doublet MIDIs	8	4	1
% of doublet MIDIs comprised of IMIDIs	25% (2/8)	25% (1/4)	0
% of doublet OMIDIs that together result in a net in-frame mutation 1	100% (8/8)	100% (3/3)	0

1 An OMIDI pair can result in a net in-frame mutation if the second OMIDI restores the reading frame and no nonsense codon occurs between the first OMIDI and the second OMIDI.

### Doublets tend to cluster in the EGFR and TP53 genes in lung cancer: the “half-life of mutation spacing” is 9 bp and 15 bp, respectively

Spontaneous *lacI* doublets (0.7% of total mutations) are clustered with a spacing fitting an exponential distribution and show a similar mutation pattern to singlets, indicating a chronocoordinate event [13], [14]. Based on the doublet spacing between two mutations, *EGFR* doublets can be divided into two groups: the proximal group (<100 bp, average spacing 22.8±24.4-bp) and the distal group (>809 bp, average spacing 12.3±5.1 kb) (Table S1). Analogous to *lacI* doublets, a subset [40% (39/98)] of *EGFR* doublets occurring in the same exon with proximal spacing shows an exponential (R^2^ = 0.9979) rather than a quasi-uniform distribution (Figure S1). Half the doublets have mutations separated by 9 bp or less (the “half-life of mutation spacing”; see Terminology in Materials and Methods).

The spacing distribution for *TP53* doublets in lung cancer occurring in the same exon (43%, 23/54) also shows proximal spacing (<86 bp, average 20.2±20.9 bp) and fits to an exponential distribution (R^2^ = 0.979) (Table S2, Figure S1) with a half-life of mutation spacing of 15 bp. Doublets occurring in different exons have distal spacing (131–4504 bp, average 1317.9±1000 bp) separated by an intron. A similar spacing to that in lung cancer was found for *TP53* doublets within the same exon in breast and colorectal cancers, with a half-life of mutation spacing of 24 and 16 bp, respectively (Tables S4 and S5).

Thus, about one-third of *EGFR* and *TP53* doublets are highly clustered (≤ 30 bp) and all doublets occurring within a single exon have an inter-mutation spacing that is exponentially distributed rather than the expected quasi-uniform distribution, consistent with chronocoordinate events in *lacI* in normal mouse tissue.

### The OMIDI pairs are strong evidence for clustered chronocoordinate mutations

The great majority of spontaneous human germlime or somatic mouse MIDIs are OMIDIs (*FIX*, Big Blue); indeed, about half are single base deletions [15]–[18]. In contrast, there is a very low fraction of OMIDIs (0.26%, 4/1526) and nonsense mutations (0.13%, 2/1526) in *EGFR* singlets, consistent with a strong selection for mutations that alter, rather than delete, function. Eight of 98 (6%) *EGFR* doublets contain a pair of frameshift mutations (OMIDI pairs; see abbreviations in Materials and Methods). All eight of the OMIDI pairs are closely spaced (Table 3, Figure S2), with the net result being an in-frame mutation. Given one OMIDI mutation, a second random mutation 3′ to the first (either IMIDI or OMIDI) will restore reading frame only 1/3 of the time, and a sub-fraction of that third is still protein truncating because a nonsense mutation occurs prior to the second mutation. In total 0.49% of reported *EGFR* mutations (8/1627) are clustered OMIDI pairs with no truncation mutation occurring before the second OMIDI. These eight OMIDI pairs are expected to reside on the same allele; otherwise, compound heterozygous OMIDI doublets will lead to two allelic truncations, eliminating all *EGFR* gene function. Meanwhile, both of the clustered OMIDIs should also occur in rapid succession (chronocoordinate mutations) within the same cell cycle because the frequency of secondary mutations randomly occurring in a proliferation-deficient cell resulting from a single OMIDI is rare. The remaining 14 deletions (in exons), 9 indels and 3 doublets are all in-frame (IMIDI) mutations (Table 2; Table S1).

**Table 3:** The characteristics of eight OMIDI paired doublets in the *EGFR* gene in lung cancer.

ID	Doublet	Exon	Spacing (bp)	Mutation Pattern	Verified Doublet Somatic Mutations	Prior to Treatment	TKI Treatment	Drug Response^1^ (Reported)	Smoking	Ethnicity	Appearence in Singlet	Verified Singlet Somatic Mutation	Singlet Drug Response^1^	PMID
5	2243_2249dup	19	2	dup7	NA	NA	NA	NA	N	Korean	N	NA	NA	17186532
	2252_2277delinsG	19		Indel25	NA			NA			N			
6	2239_2248del	19	3	del10	NA	Y	erlotinib	PR	N	NA	N	NA	NA	15329413
	2252_2256del	19		del5	NA			PR			N			
7	2235_2236delGG	19	4	del2	NA	NA	N	NA	NA	Japanese	N	NA	NA	16052218
	2241_2248delinsC	19		Indel7	NA			NA			N			
8	2235_2236delGG	19	4	del2	NA	Y	N	NA	N	Korean	N	Y	NA	17186532
	2241_2248delinsC	19		Indel7	NA			NA			N			
9	2235_2236delGG	19	4	del2	NA	Y	N	NA	Y	Korean	N	Y	NA	17186532
	2241_2248delinsC	19		Indel7	NA			NA			N			
10	2235_2236delGG	19	4	del2	NA	NA	N	NA	NA	Japanese	N	NA	NA	16052218
	2241_2251delinsC	19		Indel10	NA			NA			N			
17	2238_2247del	19	6	del10	NA	Y	gefitinib	NA	NA	Japanese	N	NA	NA	15604253
	2254_2255del	19		del2	NA			NA			N			
18	2229_2236del	19	8	del8	NA	Y	gefitinib	PR	N	NA	N	NA	PR	15329413
	2245_2252delinsT	19		Indel7	NA			PR			N		PR	

1 Drug Response: PR-partial response; SD-stable disease; PD-progressive disease.

NA: Not Available.

The mutations in the single OMIDI pair reported in the *TP53* gene are 2309 bp apart, in different exons (Table S2, ID # 14373), and, unlike the OMIDI pairs in the *EGFR* gene, do not together restore the reading frame.

### EGFR multiplets are consistent with a singlet and a highly clustered chronocoordinate second mutation

Four triplets and one quadruplet in *EGFR* were found at a frequency of 0.3% (5/1627) of lung cancers with mutations prior to treatment with tyrosine kinase inhibitor (TKI) (Table 4). It is of interest that pairs of mutations in each of the triplets and three mutations within the quadruplet are tightly clustered with proximal spacing (3–60 bp), similar to doublets in *lacI* in normal mouse tissues. The third mutation is located in a different exon with distal spacing. The data are consistent with sequential selection of two mutations in which one of the mutations is a chronocoordinate doublet or triplet.

*TP53* multiplets (6 triplets and one quadruplet) also account for 0.3% (7/2387) of total mutations (Table S3). Five multiplets (71%, 5/7) reside in the same exon or two exons, consistent with one mutation and one chronocoordinate doublet. Seven multiplets had mutations separated by 15 spacing regions. Of these, six were highly clustered (≤30 nt) and nine were within 100 nucleotides or less. Eighty percent (16/20) of multiple mutations in breast cancer and 76% (25/33) in colorectal cancer also reside in one or two exons (Tables S6, S7).

**Table 4:** The mutation characteristics and spacing within multiplets in the *EGFR* gene.

ID	Multiplets	Exon	Spacing^1^ (bp)	Het/Hom	Mutation Pattern	CpG	TS/TV	Verified as Somatic Multiple Mutation	Detected Prior to Treatment	TKI Treatment	Drug Response to multiplet 2	Smoking	Ethnicity	Appearance in Singlet	Drug Response to singlet 2 (Reported)	PMID
1	2135T>C, P712S	18	17832	Het	T>C	N	TS	NA	Y	Gefitinib	PR	Y	Taiwanese	N	NA	16870303
	**2564A>G, D855G**	**21**	38	Het	A>G	N	TS							N	NA	
	**2603A>G, E868G**	**21**		Het	A>G	N	TS							N	NA	
2	2156G>C, G719A	18	7320	NA	G>C	N	TV	NA	Y	N	NA	NA	NA	Y	PR,SD,PD	16467085
	**2327G>A, R776H**	**20**	21	NA	G>A	Y	TS							N	NA	
	**2348C>T, T783I**	**20**		NA	C>T	N	TS							Y	NA	
3	2582T>G, L861Q	21	17830	NA	T>G	N	TV	Y	Y	N	NA	NA	Australian	Y	PR,SD,PD	15741570
	**2144T>G, I715S**	**18**	14	NA	T>G	N	TV							N	NA	
	**2159C>T, S720F**	**18**		NA	C>T	N	TS							Y	PR	
4	del?^3^	19	6542	NA	del			NA	Y	N	NA	NA	NA	NA	NA	16467085
	**2305G>A, V769M**	**20**	60	NA	G>A	Y	TS							N	NA	
	**I789_L792del**	**20**		NA	del									N	NA	
5 4	2293G>A, V765M	19	6569	Het	G>A	Y	TS	NA	Y	Gefitinib	PR	N	Taiwanese	N	NA	16870303
	**2393T>A, L798H**	**20**	22	Het	T>A	N	TV							N	NA	
	**2416A>G, K806E**	**20**	24	Het	A>G	N	TS							N	NA	
	**2441T>C, L814P**	**20**		Het	T>C	N	TS							N	NA	

1 Spacing (bp) between mutations 1 and 2, followed by the spacing between mutations 2 and 3, etc.

2 Drug Response: PR-partial response; SD-stable disease; PD-progressive disease.

3 The deletion is presumed to be the most common 15 bp deletion (c2235_2249).

4 The identical set of four mutations was reported in three different patients.

**Bold Face** indicates that one mutation in the multiplet resides in a different exon relative to the other clustered mutations in the same exon.

A comparison of OMIDI pairs and closely spaced doublet and multiplet mutations in the *EGFR* and *TP53* genes is shown in Table 5. The frequency of OMIDI pairs in the *EGFR* gene is approximately 10-fold greater than in the *TP53* gene (and the one OMIDI pair in the *TP53* gene does not result in a net in-frame mutation), reflecting the more frequent selection of mutations that alter, rather than delete, EGFR protein function. The frequency of closely spaced mutations in doublets and multiplets in both genes is similar.

**Table 5:** Closely spaced doublets and multiplets detected in the *EGFR* and *TP53* genes.

	EGFR 1 (No.)	p53 1 (No.)
OMIDI pairs	0.5% (8)	0.04%(1)
Other doublets (spaced ≤30 nt)	1.4% (23)	0.8% (18)
Multiplets (at least 1 pair spaced ≤30 nt)	0.2% (3)	0.2% (4)

1 Total number of mutations: *EGFR*, n = 1627; *TP53*, n = 2387.

### All characterized EGFR doublets are allelic: a significant subset of chronocoordinate mutations provides an alternative explanation

At face value, the allelism data suggests that the driver/driver mutations in *EGFR* need to be on the same molecule [10]. However, the existence of a substantial subset of chronocoordinate mutations provides an alternative explanation. Sixteen heterozygous *EGFR* doublets from three different studies have been analyzed by cloning or allele-specific amplification (Table S1) [10], [19], [20]. Subsequent sequencing of the products revealed that all these alleles were either doubly mutated or wild type, indicating that, in every case, both mutations were located on the same allele. If the doublet pairs result from independent events, half may be expected to be compound heterozygotes. The observation of 100% allelism (16/16) vs. zero compound heterozygotes (p = 0.002) may reflect that, functionally, the driver/driver mutations are required to be on the same molecule even though the EGFR protein forms dimers [10]. However, the data are also consistent with 50% chronocoordinate events, putatively during strand replication or patch repair, and 50% independent sequential mutations without a requirement that both mutations be on the same molecule (8 of 8 allelic mutations observed when 4 of 8 are expected at random; p≤0.08).

Unfortunately, to our knowledge, allelic analysis has not been performed on heterozygous *TP53* doublets.

## Discussion

We present the first analysis of the epidemiology of doublets in lung cancer. Smoking status, race, ethnicity, gender, and age are not risk factors for doublet occurrence in lung cancer. The high frequency of clustered doublets, the exponential distribution of spacing, the occurrence of in-frame clustered OMIDI pairs, the allelic nature of doublets, and the clustering of multiplets in *EGFR* and *TP53* are consistent with a significant minority of chronocoordinate mutations in human cancer. However there are caveats for each of the above observations (see below).

We demonstrate that doublets are more frequent in the *EGFR* and *TP53* genes in human cancer than in spontaneous somatic mutations in normal mouse tissue. Within the constraints of sample size, doublet frequency does not obviously depend on smoking status, gender, ethnicity, or age. The *TP53* gene is mutated in about half of all tumors and can be used as a “mutagen test,” that is, the relative frequencies of the different types of mutation can be used as an epidemiological tool to explore the contribution of exogenous mutagens vs. endogenous processes in breast cancers [21], [22]. We hypothesized that the higher mutation rates expected in smokers would more often produce doublet mutations. However, there is no significant difference between smokers and non-smokers, consistent with doublets and multiplets arising from endogenous processes.

### Data are consistent with chronocoordinate mutations (with caveats)

Several aspects of the data are consistent with a subset of *EGFR* and *TP53* doublets arising from choronocoordinate events rather than from hypermutability (random distributed spacing) or sequential selection. About 32–35% of the doublets found in the *TP53* (35%, 19/54) and *EGFR* (32%, 31/98) genes are either OMIDI pairs or highly clustered doublets separated by ≤30 nt. However, there are caveats for each inference that must be ruled out in future work. *The main value of the present analysis is to generate data that constrain our hypotheses and, by Occam*'*s razor, generate a simple hypothesis (the subset of chronocoordinate events) consistent with multiple data.*

(1) The OMIDI pairs are the best evidence for chronocoordinate mutations. For most genes, the majority of spontaneous MIDIs are OMIDIs, but in the *EGFR* gene, OMIDIs are rare, consistent with the overall selection for altered function rather than lack of function. It is notable that the eight *EGFR* doublets containing OMIDI pairs all have closely spaced mutations that result in a net in-frame mutation, thus producing an EGFR protein with potentially altered, rather than absent, function. However, no experimental evidence of a protein containing one of these doublet mutations has been demonstrated. The most reasonable explanation for this occurrence is through chronocoordinate mutations that are selected together for altered protein function. An alternative explanation is that OMIDI pairs might somehow be sequential, although random mutations within a few nucleotides in the genome must be extremely unlikely.

(2) The relatively high frequency of clustered doublet mutations in the *EGFR* gene is consistent with chronocoordinate mutations. The exponential distribution of the spacing between the two mutations in *EGFR* and *TP53* doublets in the same exons (R^2^ = >0.98) is highly unlikely by chance.

A simulation was performed previously for each of the *TP53* exons 5–9 to test the null hypothesis that the mutations in *TP53* doublets are independent events and that the distribution of the spacing between the two mutations depends only on the spectrum of mutations in the *TP53* gene and the size of the mutation target [13]. The first mutation of each simulated doublet was drawn randomly based on the observed distribution of singlet mutations in a given exon. Using the singlet distribution from the TP53 IARC database (release 8) accounts for bias that may result from closely spaced hot spots. The second mutation in the doublet was drawn randomly based on a uniform distribution over the TP53 coding sequence for that exon, since it is likely to be a “hitchhiker” (passenger) mutation, rather than a driver mutation for the tumor.

The mutation spacing in the simulated doublets was compared with 402 actual *TP53* doublets in the IARC database from all types of cancer. The results demonstrated that two mutations within doublets are not independent events by statistical comparison of the observed and expected distributions, and indicated that more doublets occurred with a mutation spacing of less than 30 nucleotides than expected by chance (P = 0.03, 0.02, 0.0006, and 0.01 for exons 5–8). These results are consistent with the occurrence of chronocoordinate mutations in the human *TP53* gene. An alternate explanation for the clustering of doublets is that they may represent some as yet unappreciated functional constraints.

(3) The allelic nature of doublets is consistent with chronocoordinate mutations. Among 98 doublet pairs reported in the literature, only a few have been analyzed to determine whether they are present on the same or different alleles. Among 16 doublet pairs tested by cloning or allele-specific amplification, all 16 pairs were found to be present on the same allele. However, for the remaining doublet pairs, in which cloning or allele-specific amplification was not performed, the possibility that the mutations were present on different alleles cannot be ruled out.

(4) The relatively high frequency of multiplets with two or more clustered mutations and an additional mutation at a distal location also argues for a chronocoordinate mutational event plus an independent single mutational event. Again, an alternative explanation could involve functional constraints on the clustered mutations.

In aggregate, these data are consistent with a contribution of clustered chronocoordinate mutations to human cancer. An excess of doublets or multiplets with a subset of clustered distribution occurs more frequently than predicted by chance in a wide range of organisms, including riboviruses, DNA viruses, prokaryotes, yeast, and eukaryotic cell lines and tissues [23], [24].

### Data support driver plus passenger TP53 doublets in lung cancer

About 22% of the second mutations in doublets are expected to be silent if they are “passenger” mutations, rather than “driver” mutations [25]. In the *TP53* doublets, there are five silent and two intronic nucleotide changes (13%, 7/54), not significantly different from the 23.5% silent nucleotide changes expected to occur with random passenger mutations (p = 0.46) [26], [27]. Silent nucleotide changes (18%, 4/22) within *TP53* multiplets are also close to 23.5%. Together, these data imply that *TP53* doublets and multiplets consist of a driver plus passenger mutation pattern, in contrast to the driver/driver pattern found in *EGFR* doublets [10].

Doublet mutations are associated with mutation showers in mouse [9]. The advent of massively parallel sequencing can facilitate the analysis of sufficient numbers of samples to define any doublets and then determine whether these doublets are associated with mutation showers in cancer. The non-random clustering mutations in mutation showers should provide more definitive data for the occurrence of chronocoordinate mutations in human cancers. The contribution of mutation showers to cancer remains to be determined.

## Materials and Methods

### Terminology and abbreviations

#### Mutation spectrum

The relative frequencies of mutations at specific sites.

#### Mutation pattern

The relative frequencies of different types of mutations, e.g., C to T transitions vs. T to C transitions.

#### Singlet

A single mutation identified within a gene [13].

#### Tandem-base mutation (TBM)

A mutation that results in base changes at adjacent nucleotides [28], [29].

#### Doublet

Two mutations identified within a gene including a mix of TBM and non-TBM mutations.

#### Multiplet

Three or more mutations identified within a gene, excluding the situation in which all mutations are adjacent. Multiplets can include a mix of TBM and non-TBM mutations.

#### Domuplets

A mutant that is either a doublet or a multiplet. Approximately 1% of *lacI* mutant plaques are domuplets [9].

#### MIDI

Microinsertion, deletion, or indel; an insertion, deletion or indel that results in a gain or loss of 1 to 50 nucleotides [10].

#### IMIDI

In-frame MIDI

#### OMIDI

Out-of-frame MIDI

#### Half-life of mutation spacing

From the exponential fit to the data, the interval of mutation spacing corresponding to the interval encompassing half of the remaining mutations within the sample. This is analogous to the half-life of a radioisotope.

#### Chronocoordinate mutation

Multiple mutations occurring within the same cell cycle and in rapid succession, typically within seconds to minutes [13].

#### Mutation shower

Chronocoordinate multiple mutations that span multiple kilobases [9].

#### Silent mutation

A neutral mutation that does not change protein structure, including synonymous coding region changes. It is recognized that occasional mutation types overlap, e.g., a silent mutation may activate a cryptic splice site or may inactivate the normal splice site if it disrupts the splice donor consensus sequence [30]. In practice very few such overlaps were found.

### Samples and mutational analysis

In order to investigate the mechanism and characteristics of doublets and multiplets, *EGFR* mutations identified by sequencing were collected from 66 published papers and our updated *EGFR* mutation database [10], [31] (www.egfr.org). *TP53* mutations were collected from IARC version 12 (www-p53.iarc.fr) and excluded mutations in pulmonary fibrosis and in lung cancer in patients exposed to smoky coal emissions, radon, mustard gas, asbestos, heavy metals, and atomic bomb radiation (γ-rays). We also excluded two papers in which multiple mutations comprised >50% of the total mutations, most likely due to PCR artifacts, since multiple mutations generally make up only ∼3% of total mutations. *TP53* mutations in breast and colorectal cancers also were retrieved and the spacing distribution of doublets/multiplets was analyzed.

### Statistical Analysis

Mutation patterns and other categorical count distributions were tested for significant differences by the Fisher's Exact Test or unordered R×C contingency tables using the “Fisher-Freeman-Halton” test implemented by the StatXact statistical analysis software package (CYTEL Software Corporation, Cambridge, MA).

## Supporting Information

Figure S1 A subset of doublets shows proximal spacing and fits to exponential distribution in the EGFR and p53 genes in lung cancer. Panel A shows the separation (in base pairs) between the two mutations in EGFR proximal doublets (n = 37). The separation distances were divided into three groups, with spacings of 1–41 bp, 42–82 bp, and 83–123 bp, and plotted at the midpoint of each group (20, 60, and 100, respectively). Separation is defined here as the number of nucleotides between, but not including, the two mutations in a doublet. For MIDIs, separation is defined as the number of nucleotides between, but not including, the start of the first and second MIDIs. Panel B shows the spacing (in base pairs) between the two mutations in p53 proximal doublets (n = 23). The separation distances were divided into three groups, with spacings of 1–31 bp, 32–62 bp, and 63–93 bp, and plotted at the midpoint of each group (15, 30, and 45, respectively).
(0.62 MB PPT)

Figure S2 Doublets in the EGFR gene that form OMIDI pairs The wild type (wt) EGFR sequence in exon 19 from nucleotides 2227 to 2280 is shown. The eight OMIDI pairs in lung cancer are diagrammed to show the deletions (in magenta), insertions (in green) and a region that is duplicated (in yellow). Note that two of the doublets consist of two deletions each, five doublets consist of one deletion plus an indel, and one doublet has a duplication (insertion) plus an indel. In each case, the reading frame is restored (see net deletion).
(0.03 MB DOC)

Table S1 Supplementary Table 1
(0.07 MB XLS)

Table S2 Supplementary Table 2
(0.07 MB XLS)

Table S3 Supplemenatary Table 3
(0.03 MB XLS)

Table S4 Supplementary Table 4
(0.08 MB XLS)

Table S5 Supplementary Table 5
(0.11 MB XLS)

Table S6 Supplementary Table 6
(0.04 MB XLS)

Table S7 Supplementary Table 7
(0.06 MB XLS)
