# Supplementary material for: Epidemiology of Doublet/Multiplet Mutations in Lung Cancers: Evidence that a Subset Arises by Chronocoordinate Events
Source: PLoS One. 2008 Nov 13;3(11):e3714. doi: 10.1371/journal.pone.0003714 (PMC2579325; doi:10.1371/journal.pone.0003714)
Supplement: Figure S1 — A subset of doublets shows proximal spacing and fits to exponential distribution in the EGFR and p53 genes in lung cancer. Panel A shows the separation (in base pairs) between the two mutations in EGFR proximal doublets (n = 37). The separation distances were divided into three groups, with spacings of 1–41 bp, 42–82 bp, and 83–123 bp, and plotted at the midpoint of each group (20, 60, and 100, respectively). Separation is defined here as the number of nucleotides between, but not including, the two mutations in a doublet. For MIDIs, separation is defined as the number of nucleotides between, but not including, the start of the first and second MIDIs. Panel B shows the spacing (in base pairs) between the two mutations in p53 proximal doublets (n = 23). The separation distances were divided into three groups, with spacings of 1–31 bp, 32–62 bp, and 63–93 bp, and plotted at the midpoint of each group (15, 30, and 45, respectively). (0.62 MB PPT) [file pone.0003714.s001.ppt]

## Slide 1
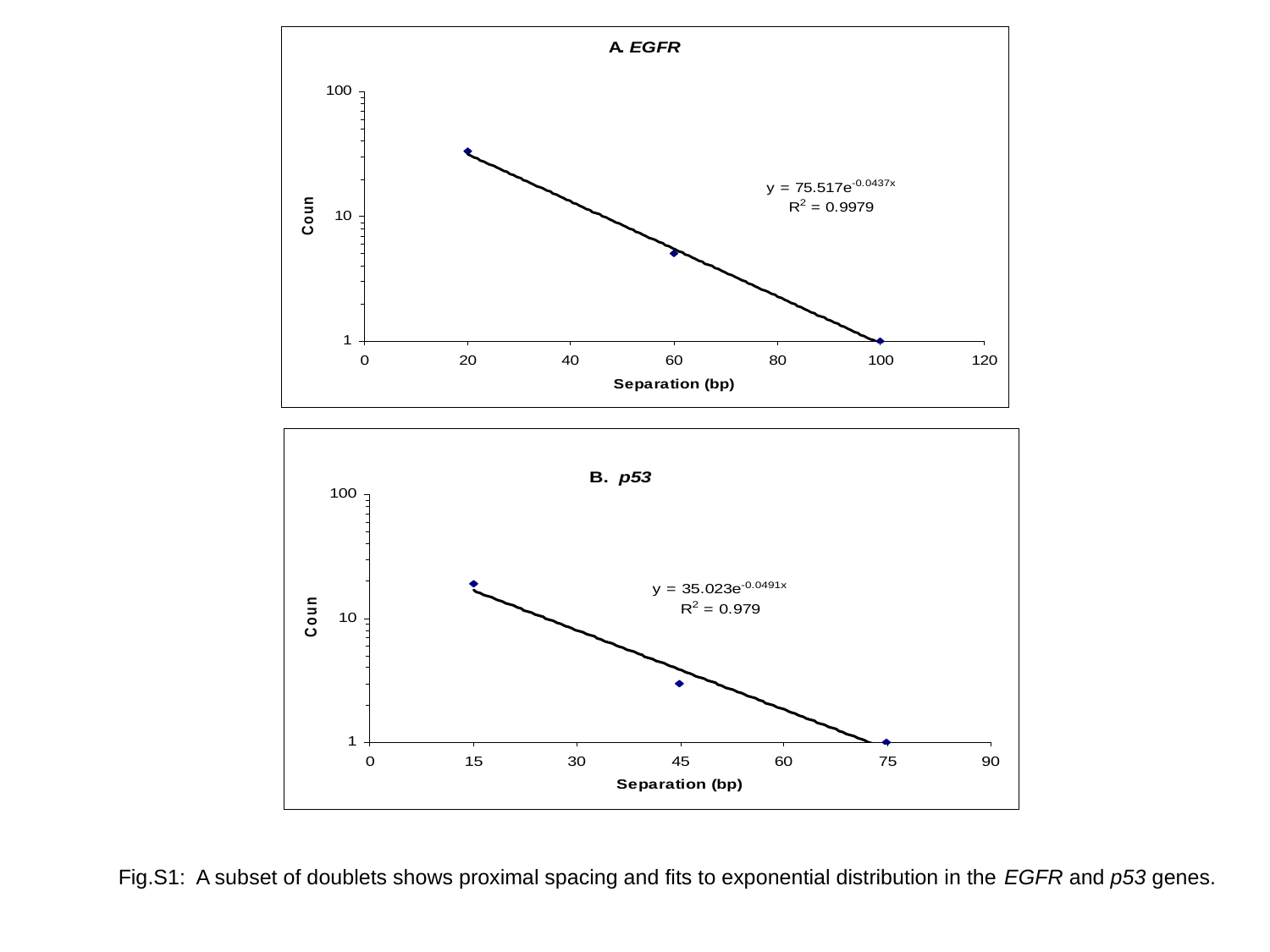

Fig.S1: A subset of doublets shows proximal spacing and fits to exponential distribution in the EGFR and p53 genes.
