# Supplementary material for: Epidemiology of Doublet/Multiplet Mutations in Lung Cancers: Evidence that a Subset Arises by Chronocoordinate Events
Source: PLoS One. 2008 Nov 13;3(11):e3714. doi: 10.1371/journal.pone.0003714 (PMC2579325; doi:10.1371/journal.pone.0003714)
Supplement: Figure S2 — Doublets in the EGFR gene that form OMIDI pairs The wild type (wt) EGFR sequence in exon 19 from nucleotides 2227 to 2280 is shown. The eight OMIDI pairs in lung cancer are diagrammed to show the deletions (in magenta), insertions (in green) and a region that is duplicated (in yellow). Note that two of the doublets consist of two deletions each, five doublets consist of one deletion plus an indel, and one doublet has a duplication (insertion) plus an indel. In each case, the reading frame is restored (see net deletion). (0.03 MB DOC) [file pone.0003714.s002.doc]

Figure S2. Doublets in the EGFR gene that form OMIDI pairs

**NT NT NT**

**2227 ‌‌ 2259 2280**

wt: GCTATCAAGGAATTAAGAGAAGCAACATCTCCGAAAGCCAACAAGGAAATCCTC

18: GCTATGAAGGAATTAAGAGAAGCAACATCTCCG del8 + del8/ins1 ► Net del 15

T

7,8 GCTATGAAGGAATTAAGAGAAGCAACATCTCCG del2 + del8/ins1 ► Net del 9

& 9: C

10: GCTATGAAGGAATTAAGAGAAGCAACATCTCCG del2 + del11/ins1 ► Net del 12

C

17: GCTATGAAGGAATTAAGAGAAGCAACATCTCCG del10 + del2 ► Net del 12

6: GCTATGAAGGAATTAAGAGAAGCAACATCTCCG del10 + del5 ► Net del 15

5: GCTATCAAGGAATTAAGAGAAGCAACATCTCCGAAAGCCAACAAGGAAATCCTC

**^** G

GAGAAGC

dup (ins)7 + del26/ins1 ► Net del 18

‌The wild type (wt) *EGFR* sequence in exon 19 from nucleotides 2227 to 2280 is shown. The eight OMIDI pairs are diagrammed to show the deletions (in magenta), insertions (in green) and a region that is duplicated (in yellow). Note that two of the doublets consist of two deletions each, five doublets consist of one deletion plus an indel, and one doublet has a duplication (insertion) plus an indel. In each case, the reading frame is restored (see net deletion).
